# Investigating hormone-induced changes in affective state using the affective bias test in male and female rats

**DOI:** 10.1016/j.psyneuen.2020.104647

**Published:** 2020-05

**Authors:** Justyna K. Hinchcliffe, Michael Mendl, Emma S.J. Robinson

**Affiliations:** a University of Bristol, School of Physiology, Pharmacology & Neuroscience, Biomedical Sciences Building, University Walk, Bristol, BS8 1TD, UK; b School of Veterinary Sciences, University of Bristol, Langford House, Langford, Bristol BS40 5DU, UK

**Keywords:** Affective bias, Oestradiol, Carbetocin, Testosterone, Desmopressin, Progesterone

## Abstract

Recent clinical and pre-clinical research suggests that affective biases may play an important role in the development and perpetuation of mood disorders. Studies in animals have also revealed that similar neuropsychological processes can be measured in non-human species using behavioural assays designed to measure biases in learning and memory or decision-making. Given the proposed links between hormones and mood, we used the affective bias test to investigate the effects of different hormone treatments in both male and female rats. Animals were pre-treated with acute doses of hormone or vehicle control prior to learning each of two independent substrate-reward associations. During a subsequent choice test, positive or negative biases were observed by animal’s preference towards or away from the substrate learnt during drug treatment respectively. In both sexes, oestradiol and the oestrogen-like compound bisphenol A induced positive biases, whilst blockade of oestrogen hormones with formestane induced a negative bias. Progesterone induced a negative bias in both sexes, but testosterone only induced a negative bias in males. Blocking testosterone with flutamide induced a positive bias in both sexes at the higher dose (10 mg/kg). The oxytocin analogue, carbetocin induced positive biases in both sexes but the vasopressin analogue, desmopressin, induced a positive bias in male rats only. These results provide evidence that modulating levels of hormones using exogenous treatments can induce affective biases in rats. They also suggest that hormone-induced affective biases influence cognitive and emotional behaviour and could have longer-term effects in some mood disorders.

## Highlights

• The affective bias test provides new insights into the relationship between mood-related hormones and affective biases in rats.
• Treatment with oestradiol, bisphenol A and carbetocin induced significant positive affective biases in both male and female rats, whilst desmopressin induced a positive affective bias in males only.
• The opposite effects to oestradiol were found following formestane treatment, inducing a negative affective bias in both sexes.
• In male rats, a negative affective bias was developed following treatment with the low dose of progesterone, whilst in females following the higher dose of progesterone.
• Testosterone induced a dose-dependent negative affective bias in males, whilst in females there was no effect found. A positive affective bias was developed in both groups following treatment with the high dose of flutamide.

## 1 Introduction

Recent findings suggest that affective biases (the process whereby cognitive functions such as learning and memory and decision-making are modified by emotional state), may play an important role in the cause and treatment of depression (Harmer et al., 2017; Robinson, 2018). It is hypothesised that negative affective biases contribute to the reinforcement of negative feelings and beliefs in depression (Disner et al., 2011). Antidepressants have been shown to induce positive affective biases which is thought to influence the reversal of symptoms of depression over time (Harmer et al., 2017). Studies in both human and non-human species have shown that affective biases can influence many different cognitive domains (Roiser et al., 2012; Robinson, 2018). These include findings that people with anxiety and/or depression tend to exhibit biases in attention, memory and decision-making particularly in relation to ambiguous information or future events (Mogg et al., 2006; Roiser et al., 2012). Most studies in humans investigate the processing of emotional information and show that depression is associated with reduced positive and/or enhanced negative biases in emotional interpretation and emotional memory (Roiser et al., 2012). For example, depressed patients show greater recall of negative stimuli and exhibit faster reaction times to sad faces versus happy faces (Disner et al., 2011; Elliott et al., 2011; Roiser et al., 2012). Antidepressant treatments have also been shown to positively bias emotional processing in both healthy volunteers and patients which importantly, can occur after acute or short-term treatment and before subjective changes in mood become apparent (Mogg et al., 2006; Roiser et al., 2012). Building on these empirical observations and underlying neuropsychological hypotheses, tasks have been developed for non-human species which may facilitate quantification of similar affective biases (Mendl et al., 2009; Hales et al., 2014; Robinson, 2018).

Since the first report of an affective bias task for rats (Harding et al., 2004), two types of behavioural assay for non-human species have been developed. The judgement bias task (also called the ambiguous cue interpretation task) is designed to quantify affective biases in a two-choice decision-making task (for review see, Hales et al., 2014). Animals are first trained to associate two distinct cues with positively or negatively-valenced outcomes. Biases in decision-making are then quantified by presenting the animals with intermediate, ambiguous cues and recording their responses. More positive affective states are associated with a greater number of responses in anticipation of positive outcomes during ambiguous cue presentation. Studies using pharmacological and phenotypic models suggest that these biases are generally consistent with predicted changes in the underlying affective state, although there are exceptions (for review and more detailed discussion see, Hales et al., 2014). The affective bias test (ABT), first reported in 2013, is designed to quantify biases linked to learning and memory (for review see, Hales et al., 2014; Robinson, 2018). The assay uses a within-subject study design where animals encounter two distinct and independent learning experiences under treatment or control conditions. The value of the experiences is kept constant and, using a choice test, biases resulting from the treatment can be quantified. Treatments inducing a positive bias result in a preference for the treatment-paired experience, whilst negative biases are observed as a preference for the control paired experience. The ABT has been extensively validated using pharmacological, psychosocial and neurobiological studies with results consistent with predicted effects on affective state (Stuart et al., 2013, 2015; Hinchcliffe et al., 2017; Stuart et al., 2017).

In this study, we used the ABT to investigate the effects of the gonadal hormones, oestrogen, progesterone and testosterone, and peptide hormones, oxytocin and vasopressin on biases in learning and memory in both male and female rats. The gonadal hormones and the peptide hormones have been linked to changes in emotional processing and mood (Swaab et al., 2005; Neumann and Landgraf, 2012; Backstrom et al., 2014; McHenry et al., 2014). These hormones play important roles in modulating normal sexual and social behaviour but, may also contribute to the development of mood-related symptoms in mental health disorders, and the emotional symptoms associated with premenstrual, postpartum and menopausal disorders (Backstrom et al., 2014; Swaab et al., 2005). Previous studies suggest that elevated oestradiol is associated with more positive affective states including reduced anxiety and antidepressant effects (Albert and Newhouse, 2019). In contrast, progesterone has been linked to low mood, and induction of premenstrual syndrome or premenstrual dysphoric disorder (Swaab et al., 2005; Backstrom et al., 2014). The effects of testosterone in a rodent model of depression suggested an antidepressant effect (Buddenberg et al., 2009). On the other hand, high levels of testosterone increase aggression behaviours in men and in male rats (Finkelstein et al., 1997; Rosell and Siever, 2015). Testosterone can also modulate the stress response, although with different outcomes reported. For example, some studies report increased responsiveness (Mehta et al., 2008), whilst others suggest a reduction in the stress response (Stephens et al., 2016). Although previous studies have shown links between hormone levels and emotional behaviour, their effects on affective biases is unknown. There have also been issues in the interpretation of previous studies in rodent models of depression given the limitations associated with these approaches (Slattery and Cryan, 2017). In particular, conventional methods of assessing emotional behaviour in rodents have limited translational validity and generally rely on stress related methods. Furthermore, there is a lack of consistency in terms of the depression-like phenotype which develops in males versus females which do not necessarily reflect sex differences in clinical populations (Kokras and Dalla, 2014). Thus, to further understand the effects of the gonadal hormones on affective biases, we tested the both androgen hormones and androgen receptor antagonists, flutamide, and aromatase inhibitor, formestane. There is also a detailed literature investigating the role of oxytocin and vasopressin and how they contribute to emotional behaviour (Neumann and Landgraf, 2012). It has previously been reported that oxytocin has antidepressants effects in the forced swim test (FST) (Balmus et al., 2018). In contrast, vasopressin neurons in the paraventricular nucleus are activated in depressed patients and have functional consequences for hypothalamic-pituitary-adrenal (HPA) axis responsivity (Neumann and Landgraf, 2012). Studies in rodents suggest that vasopressin V1b antagonists have both anxiolytic and antidepressant-like effects (Iijima et al., 2014). Due to the short half-lives and poor bioavailability of the peptide hormones, we tested the stable analogues carbetocin and desmopressin (Broadbear et al., 2014). We also tested the proposed endocrine disruptor bisphenol A which may mimic oestradiol and is a common component of everyday items e.g. plastic bottles, water pipes, CDs etc. (see report, EFSA panel, 2015). These results provide new insights into how these hormones modulate affective biases and illustrate possible implications for effects on mood considering the neuropsychological hypothesis of depression (Robinson, 2018).

## 2 Methods

### 2.1 Animals and housing

Subjects were twelve male and twelve female Sprague Dawley rats (Charles River, UK) run as independent cohorts. Males weighed approximately 300−350 g and females weighed 200−250 g at the start of experimental manipulations. Animal weights were checked daily and their growth monitored weekly against a standard curve for Sprague Dawley rats. All animals were housed in same-sex pairs in enriched laboratory cages (55 × 35 × 21 cm) with sawdust, paper bedding, cotton rope, cardboard tubes and red Perspex houses (30 × 17 × 10 cm), in temperature-controlled conditions (21 ± 1 °C) and under a 12:12 h reverse light–dark cycle (lights off at 07:00 h). Rats were mildly food restricted to approximately 90 % of their free feeding weights (∼18 g of food per rat/day laboratory chow (Purina, UK)). Female rats were housed in a room with male animals to help maintain a normal oestrus cycle. Water was freely available, except during the pairing and test sessions in the affective bias test and the consumption test. The behavioural procedures and testing were performed during the animals’ active phase between 09:00 h and 17:00 h. All experimental procedures were conducted in accordance with the UK Animals (Scientific Procedures) Act 1986 and were approved by the local ethical review group (University of Bristol).

### 2.2 Affective bias test (ABT)

#### 2.2.1 General protocol

All testing was carried out in a perspex arena (40 × 40 cm) with two ceramic bowls (Ø 10 cm) and a range of digging substrates (reward-paired substrates - ‘CS + A’ or ‘CS + B’ versus unrewarded substrate – ‘CS-’, matched for digging effort, see supplementary materials, Table S1). The experimental training and testing for ABT were similar to those previously described (Stuart et al., 2013, 2015; Hinchcliffe et al., 2017). The training protocol consisted of habituation to the arena and three digging training sessions (20 trials per session) with a bowl filled with increasing amounts of digging substrate (sawdust) to obtain food reward (one 45 mg purified rodent tablet, Test Diet, Sandown Scientific, UK, catalogue number #1811155, containing sucrose, casein, maltodextrin, corn starch, corn oil, minerals, vitamins, magnesium stearate, DL-methionine). Once each animal was able to find the food pellet within 30 s on 10 consecutive trials, the digging training was complete. On the last day of training, animals underwent a discrimination session allowing them to explore two bowls with two novel digging substrates (reward-paired substrate, CS+, with single pellet versus unrewarded substrate CS-). A reward pellet was crushed into the bowl and mixed within the unrewarded substrate, to avoid choices based on odour. On each trial, the animal was individually placed in front of the two bowls. Once the animal made a choice and started digging in one bowl, the other bowl was removed by the experimenter. Choice of the reward-paired substrate was marked as a ‘correct’ trial, digging in the CS- substrate was classified as an ‘incorrect’ trial and if an animal failed to approach and explore the bowls within 30 s, the trial was recorded as an ‘omission’. Trials were continued until the rat achieved six consecutive correct choices for the reward-paired substrate. All animals completed training and were included in the subsequent studies (n = 12 per cohort).

#### 2.2.2 Hormone dose-response studies

All studies were based on four pairing sessions (one per day) followed by a choice test on the fifth day of that week. Each pairing session followed the same procedures as the discrimination session detailed above. During four pairing sessions, each animal learnt to associate two different digging substrates with acquiring a food reward under vehicle or treatment conditions. Each trial involved a choice between two bowls containing two different digging substrates, one reward-paired (‘CS + A’ or ‘CS + B’, containing a single reward pellet, counterbalanced by treatment) and the other an unrewarded CS- substrate. The CS- digging substrate was kept the same for the four pairing sessions and a reward pellet was crushed into the bowl and mixed within the substrate. CS + A was either presented on days 1 and 3 of the pairing sessions or on days 2 and 4, and CS + B was presented on the other two days. This was counterbalanced for animals and treatment. Presentation of one of these substrates (the ‘treatment-paired substrate’) was associated with administration of the drug treatment whilst the other was associated with vehicle treatment. The value of each experience was equal (one reward pellet) and all factors (i.e. bowl location, substrates, pairing sessions, hormone treatments) were fully counterbalanced. The number of trials to reach the discrimination criterion on each day, and response latency to dig were recorded for each animal.

Affective biases were quantified during the choice test on day 5 in which the two previously rewarded substrates (‘CS + A’ and CS+‘B’) were presented at the same time for 30 trials. Trials were reinforced using a random schedule with a single-pellet reward baited in either bowl with a probability of one in three. Both bowls contained a crushed pellet to reduce the likelihood of the animal using odour to find the reward. The animals’ choices and latency to dig were recorded.

### 2.3 Consumption test

To assess whether gonadal hormone treatments have any effects on appetite and food intake, we used a consumption test where the quantity of food (reward pellets, 45 mg pellets, Test Diet, UK) consumed by an animal within 10 min was measured. To match experimental conditions with the ABT, animals subjected to this consumption test were mildly food restricted and hormones were injected 30 min prior to testing. The consumption test was carried out in the ABT arena with one pottery bowl (Ø 5 cm). The study took place over four non-consecutive days using a fully counterbalanced design with the dose of each hormone that induced the largest affective bias being administered on each day followed by a food consumption test. Doses used were: oestradiol (males 1.0 μg/kg, females 10.0 μg/kg), progesterone (males 1.0 mg/kg, females 10.0 mg/kg), testosterone (males 10.0 mg/kg, females 10.0 mg/kg) and vehicle. Each animal was placed in the arena with the bowl containing 50 g of food reward pellets. Once the Perspex lid of the arena was closed, the test began. After 10 min, the rat was put back into the home cage and the uneaten food recovered, weighed and recorded. The bowl was re-baited for each animal. Absolute consumption was calculated as weight (in grams) of food left in the bowl subtracted from total weight of food placed in the bowl. The rats were weighed before every day of testing to allow calculation of consumption relative to body weight (grams/kilograms).

### 2.4 Drugs

Oestradiol (17β-estradiol; 1.0, 10.0 μg/kg, administered subcutaneously: SC, t=-30 min), progesterone (4-Pregnene-3,20-dione; 1.0, 10.0 mg/kg, SC, t=-30 min), testosterone (1.0, 10.0 mg/kg, SC, t=-30 min), formestane (1.0, 10.0 mg/kg, SC, t=-30 min), flutamide (1.0, 10.0 mg/kg, administered orally: PO, t=-30 min), bisphenol A (0.05, 0.5 mg/kg, PO, t =-30 min), carbetocin (carbetocin acetate; CBT; 0.3 mg/kg, SC, t=-15 min), and desmopressin ([deamino-Cys1, d-Arg8]-Vasopressin acetate salt hydrate; DDAVP; 0.1 mg/kg, SC, t=-15 min) were purchased from Sigma-Aldrich, UK. Choice of doses was based on previous studies (Swaab et al., 2005; Neumann and Landgraf, 2012; Backstrom et al., 2014; McHenry et al., 2014). Single low doses of desmopressin has been found to activate the HPA axis and lead to cortisol release in human participants suggesting it crosses the blood brain barrier (Scott et al., 1999). Vehicle solution for oestradiol, progesterone, testosterone and formestane was 5% DMSO and 95 % sesame oil; for flutamide and bisphenol A was 1% ethanol in strawberry milkshake, and for carbetocin and desmopressin was saline. Prior to the start of the experiments, rats were trained to drink milkshake (Frijj, UK, 0.5 ml) from a 1 ml syringe to facilitate oral drug dosing. On each day of treatment, drugs and vehicle solutions were freshly prepared. All studies used a within-subject design and there was a minimum of 7 days drug free before commencing a new treatment. All subcutaneous injections were performed with minor animal restraint and injected on their left or right flank (changing daily) to minimise the stress associated with restraint. All experiments were carried out with the experimenter blind to treatment.

## 3 Data Analysis

Data were analysed and the graphs were created using GraphPad Prism 6.0 (GraphPad Software, USA). Choice bias was calculated as the number of choices made for the treatment-paired substrate divided by the total number of trials (treatment-paired substrate + vehicle-paired substrate) multiplied by 100 to give a percentage value. A value of 50 was then subtracted to give a % choice bias score where a bias towards the treatment-paired substrate gave a positive value and a bias towards the vehicle-paired substrate gave a negative value.

The % choice bias results from the dose response studies were analysed using a repeated measures ANOVA with dose (oestradiol, formestane, bisphenol A, progesterone, testosterone and flutamide study) or treatment (carbetocin and desmopressin study) as the within-subject factor, and one-sample *t*-test against the null hypothesised mean of 0% choice bias as post-hoc tests. A Shapiro-Wilk test was used to determine a normal distribution, the Huynh-Feldt correction was used to adjust for violations of the sphericity assumption, and Levene’s test was used to correct for inequality of variances for the % Choice bias. A repeated measures ANOVA with treatment as the within-subject factor was used to analyse the results from consumption test. Analysis of the trials to criterion and response latency utilised a paired *t*-test, comparing vehicle vs treatment for each animal during the pairing sessions.

## 4 Results

### 4.1 Effects of acute hormone manipulations on affective biases in male and female rats

**Fig. 1:**
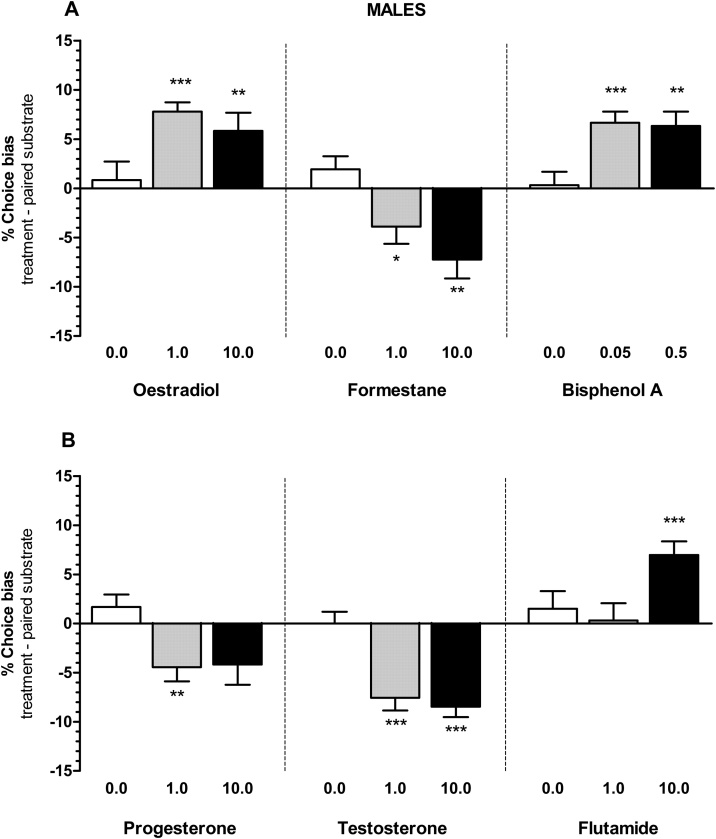
The effects of hormonal manipulations on % choice bias in male rats. These results illustrate the positive and the negative affective biases induced by acute treatment with oestradiol, formestane, bisphenol A (panel A) and progesterone, testosterone, flutamide (panel B) in male rats (n = 12). Data shown as mean % choice bias ± SEM, *p < 0.05, **p < 0.01, ***p < 0.001, one sample *t*-test against a null hypothesised mean of 0% choice bias.

**Fig. 2:**
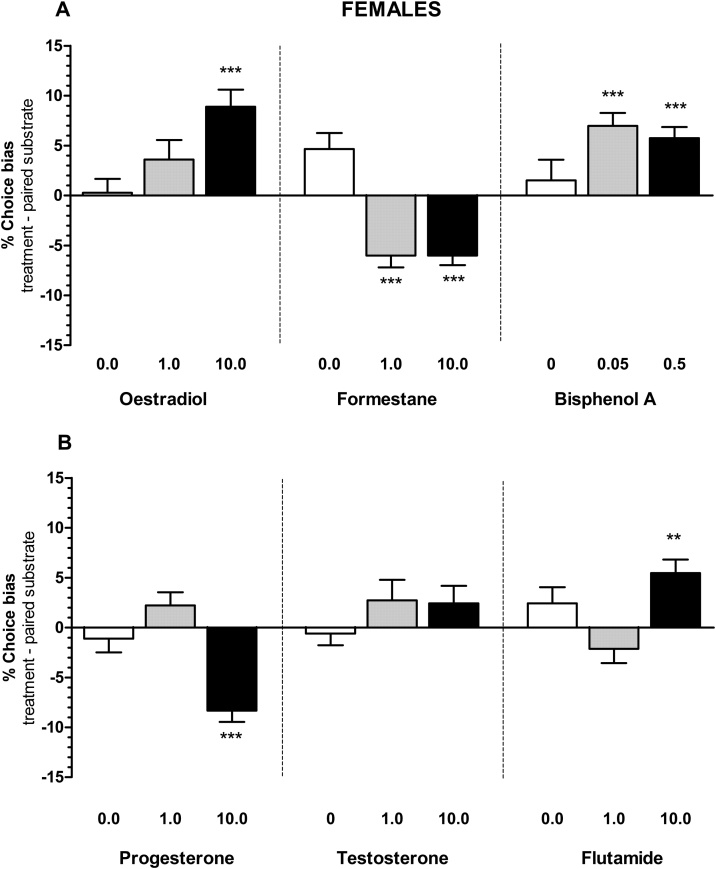
The effects of hormonal manipulations on % choice bias in female rats. The results illustrate the positive and the negative affective biases induced by acute treatment with oestradiol, formestane, bisphenol A (panel A) and progesterone, testosterone, flutamide (panel B) in female rats (n = 12). Data shown as mean % choice bias ± SEM, *p < 0.05, **p < 0.01, ***p < 0.001, one sample *t*-test against a null hypothesised mean of 0% choice bias.

Animals made significantly more choices for the substrate-reward association learnt following acute treatment with female gonadal hormone, oestradiol (males, 0.0–10.0 μg/kg, RM ANOVA F_2,11_ = 4.033, p = 0.0322, Fig. 1A and females, 0.0–10.0 μg/kg, RM ANOVA F_2,11_ = 7.005, p = 0.0044, Fig. 2A), endocrine disruptor, bisphenol A (males, 0.0-0.5 mg/kg, RM ANOVA F_2,9_ = 6.339, p = 0.0082, Fig. 1A and females, 0.0-0.5 mg/kg, RM ANOVA F_2,10_ = 4.085, p = 0.0325, Fig. 2A), and androgen receptor antagonist, flutamide (males, 0.0–10.0 mg/kg, RM ANOVA F_2,10_ = 4.593, p = 0.0228, Fig. 1B and females, 0.0–10.0 mg/kg, RM ANOVA F_2,10_ = 7.100, p = 0.0047, Fig. 2B) indicating positive affective biases. Positive biases were observed for both 1.0 and 10.0 μg/kg doses of oestradiol in males (one sample *t*-test, t_11_= 8.208, p < 0.0001 and t_11_= 3.169, p = 0.0089 respectively) and 10 μg/kg in females (one sample *t*-test, t_11_= 5.204 p = 0.0003) with a trend towards an effect at 1 μg/kg (one sample *t*-test, t_11_= 1.857, p = 0.09), for both 0.05 mg/kg and 0.5 mg/kg doses of bisphenol A in males (one sample *t*-test, t_9_= 6.000, p = 0.0002 and t_9_ = 4.385, p = 0.0018 respectively) and in females (one sample *t*-test, t_10_ = 5.333 p = 0.0003 and t_10_= 5.190, p = 0.0004 respectively), but only for the higher dose of 10 mg/kg of flutamide in males (one sample *t*-test, t_10_ = 5.043, p = 0.0005) and in females (one sample *t*-test, t_10_ = 3.985, p = 0.0026).

Progesterone treatment in males (0.0–10.0 mg/kg, RM ANOVA F_2,11_ = 4.592, p = 0.0215, Fig. 1B) induced a negative bias effect only at a low dose of 1.0 mg/kg (one sample *t*-test, t_11_ = 3.084, p = 0.0104), whilst in females (0.0–10.0 mg/kg, RM ANOVA F_2,11_ = 19.70, p = 0.0001, Fig. 2B) only at a high dose of 10.0 mg/kg (one sample t-test, t_11_ = 7.416, p < 0.0001).

**Fig. 3:**
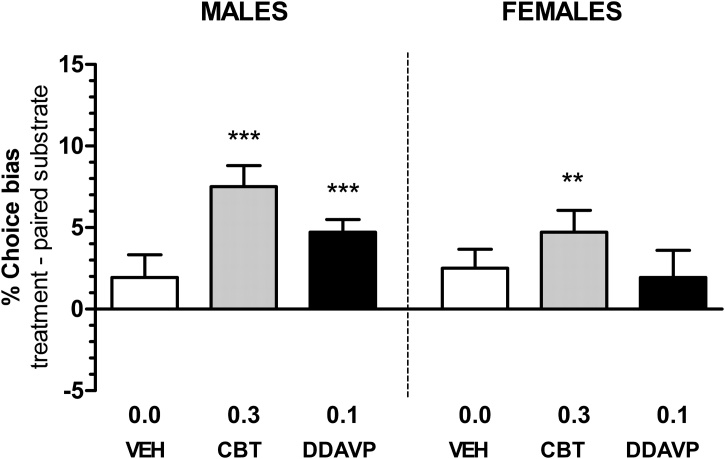
The effects of carbetocin and desmopressin on % choice bias in male and female rats. Both male and female rats (n = 12 each) showed significant positive affective biases following acute treatment with oxytocin stable analogue, carbetocin (CBT). The graphs also demonstrate induction of positive affective bias in male rats following acute vasopressin stable analogue, desmopressin (DDAVP) treatment. Vehicle (VEH) treatment did not produce any effects. Data shown as mean % choice bias ± SEM, *p < 0.05, **p < 0.01, ***p < 0.001, one sample *t*-test against a null hypothesised mean of 0% choice bias.

Acute treatment with the male gonadal hormone, testosterone induced a negative bias in males only (0.0–10.0 mg/kg, RM ANOVA F_2,10_ = 19.80, p = 0.0001, Fig. 1B) for both 1.0 mg/kg and 10.0 mg/kg doses of testosterone (one sample *t*-test, t_10_ = 5.926, p = 0.0001 and t_10_ = 8.150, p < 0.0001 respectively). Testosterone treatment in females did not show any effects (0.0–10.0 mg/kg, RM ANOVA F_2,10_ = 0.9265, p = 0.4123, Fig. 2B). The aromatase inhibitor, formestane induced positive biases in both sexes (males, 0.0–10.0 mg/kg, RM ANOVA F_2,11_ = 6.897, p = 0.0047, Fig. 1A and females, 0.0–10.0 mg/kg, RM ANOVA F_2,10_ = 18.58, p = 0.0001, Fig. 2A), and with effects observed for both 1.0 and 10.0 mg/kg doses of formestane in males (one sample *t*-test, t_11_= 2.244, p = 0.0463 and t_11_ = 3.736, p = 0.0031 respectively) and females (one sample *t*-test, t_9_= 5.014, p = 0.0007 and t_9_= 6.194, p = 0.0002 respectively),

Oxytocin and vasopressin analogues induced positive biases in male rats (RM ANOVA, F_2,33_ = 5.491, p = 0.0090, Fig. 3, carbetocin 0.3 mg/kg, one sample *t*-test, t_11_ = 5.745, p = 0.0001, Fig. 3) and desmopressin (0.1 mg/kg, one sample *t*-test, t_11_ = 6.189, p < 0.0001, Fig. 3). Female rats (RM ANOVA, F_2,33_ = 1.099, p = 0.3452, Fig. 3) treated with the oxytocin analogue developed a positive bias (one sample *t*-test, t_11_ = 3.559, p = 0.0045), while vasopressin analogue showed no effect.

There were no significant effects of treatment during pairing sessions, either on response latency or number of trials to criterion following all hormone manipulations (see Supplementary materials, Table S2 and S3).

### 4.2 Consumption test

**Fig. 4:**
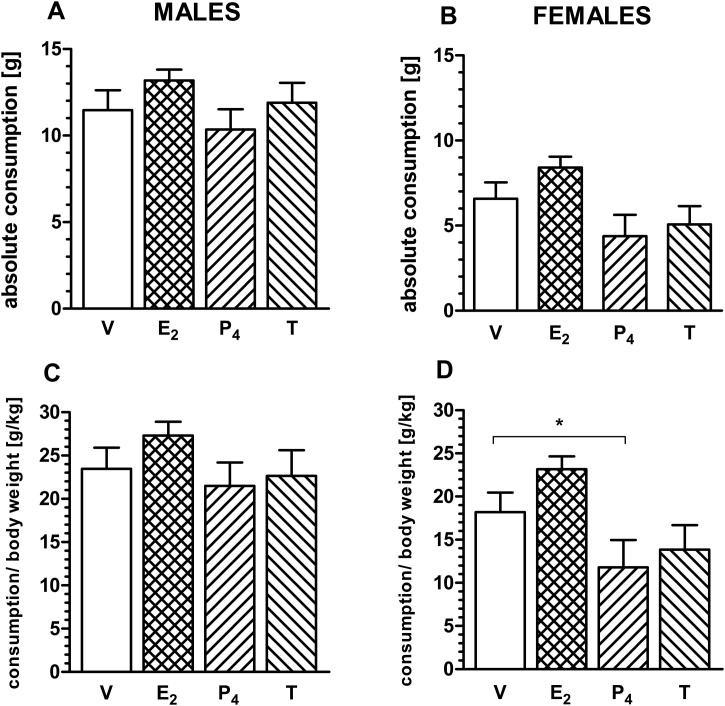
The effects on food intake following oestradiol, progesterone, testosterone and vehicle administration in male (panel A and C, n = 12) and female rats (panel B and D, n = 12). Data shown as an absolute consumption [g] and consumption per body weight [g/kg] ± SEM; oestradiol (E_2_), progesterone (P_4_), testosterone (T), vehicle (V); *p < 0.05.

In the voluntary pellet consumption test male animals under different treatments did show significant main effect in the absolute consumption (RM ANOVA F_3,33_ = 4.450, 0.010, Fig. 4A) but did not in the absolute consumption per body weight [g/kg] (RM ANOVA F_3,33_ = 2.823, 0.054, Fig. 4C). Study using female animals shown main effects in the absolute consumption (RM ANOVA F_3,30_ = 7.785, p = 0.0005, Fig. 4B) and in the absolute consumption per body weight [g/kg] (RM ANOVA F_3,30_ = 8.132, p = 0.0004, Fig. 4D). Post hoc Dunnett’s test revealed no significant differences in both males and females in the absolute consumption between the vehicle and hormone treatments, and also in the absolute consumption per body weight in males. The only changes were found in females between the vehicle and progesterone in the absolute consumption per body weight. Females consumed less reward pellets after progesterone treatment than vehicle (p = 0.045, Fig. 4D).

## 5 Discussion

Our studies have shown that acute hormone treatments can induce affective biases in male and female rats. The effects, in terms of induction of a positive or negative bias, were similar for both sexes but with some exceptions. In both sexes, treatment with oestradiol and bisphenol A and the oxytocin analogue, carbetocin, induced a positive bias. The vasopressin analogue, desmopressin, induced a positive bias but only in male rats. The lower dose of progesterone induced a negative bias in male rats, but only the higher dose was significantly effective in female rats. Testosterone treatment induced a negative bias in male rats but had no effect in females. When the effects of the gonadal hormones were blocked, the opposite effects were seen. Both the low and high dose of formestane induced a negative bias in male and female rats. Interestingly, despite the lack of effect of testosterone in the female animals, flutamide induced positive biases in both sexes. Data from pairing sessions indicated no effects of treatment on the number of trials to reach criterion or latency to dig in the bowls under all experimental manipulations. None of the compounds induced non-specific effects (e.g. motivation changes, sedation) in animals compared to vehicle treatments. Consumption tests in both male and female rats did not reveal any clear changes in appetite when compared with vehicle treatment although there were main effect findings with the ANOVA. The only change was found in females’ consumption per body weight, who consumed less reward pellets after progesterone treatment than vehicle.

Oestradiol treatment induced a positive bias in male and female rats, whilst blocking its effects using formestane induced a negative bias. These effects are consistent with those observed in conventional models of depression where oestradiol treatment has an antidepressant effect in the FST in intact animals (Walf and Frye, 2005) and gonadectomized animals (Carrier et al., 2015). Previous studies in humans have found that high levels of oestradiol can alter emotional learning by protecting hippocampal activity and its plasticity and reducing the psychological effects of stress (see review, Albert and Newhouse, 2019). Oestradiol has also been shown to have effects on emotional memory, by attenuating recall of negative emotional stimuli (Wegerer et al., 2014). In rats, intra-amygdala infusions of oestradiol reduced depressive behaviours in the FST (Frye and Walf, 2004). Previous studies found that the amygdala plays a crucial role in development of the affective biases (Stuart et al., 2015) suggesting this may be an important locus for the effects observed with oestradiol in these experiments. It has also been suggested that elevated levels of oestrogen modulate HPA axis activity and reduce the physiological and psychological stress response (Albert and Newhouse, 2019). Interestingly, we also observed positive biases in rats treated with bisphenol A. BPA binds to both alpha and beta oestrogen receptors, and therefore can mimic oestrogens effects (EFSA report, 2015). In the ABT the effects of BPA are similar to those seen with oestradiol suggesting that acute exposure to these levels of the compound can have oestrogen-like effects on emotional behaviour. It should be noted that doses of 50 μg/kg and 500 μg/kg were used in this study based on the guidelines in the European Food Safety Authority (EFSA) report, which recommended a Tolerable Daily Intake (TDI) of 50 μg/kg (EFSA report, 2015). However, in 2017 EFSA changed this to 4 μg/kg.

To further investigate the effects of endogenous oestradiol we used, formestane, a selective aromatase inhibitor. Acute administration of formestane induced a dose-dependent negative bias, the opposite effect to oestradiol treatment suggesting inhibition of endogenous oestradiol has negative effects on emotional behaviour in both sexes. It previously has been shown that formestane inhibits oestrogen synthesis in the brain, and by inhibiting the conversion of androgens into oestrogens (Dowsett, 1994). Acute formestane treatment lacks effects in FST (Martinez-Mota et al., 2008), but does attenuate antidepressant-like actions of fluoxetine and desipramine suggesting oestradiol may contribute to these drugs antidepressant effects (Martinez-Mota et al., 2008). Previous studies in the ABT have shown that acute treatments which induce a negative bias also have higher levels of pro-depressant effects in man and can cause impairments in reward processing following chronic treatment (Stuart et al., 2017). Formestane was used in the treatment of breast cancer and these data suggest that long term use may have caused mood related side effects (Wiseman and Goa, 1996).

Acute treatment with progesterone induced a negative bias in the ABT consistent with its predicted effects on mood and association with negative emotional states including irritability, aggression and the development of premenstrual syndrome or postnatal depression (Swaab et al., 2005; Backstrom et al., 2014). In the forced swim test, progesterone administered at similar doses caused an increase in immobility time, suggesting a pro-depressant-like effect in mice (Kaur and Kulkarni, 2002). Progesterone can also have effects through its active metabolite, allopregnanolone which can act indirectly as a modulator of the GABA_A_ receptor complex and induce negative effects on emotional behavior (Backstrom et al., 2014). Interestingly, there have been some discrepancies in the literature in relation to progesterone’s effect on mood (see review, Backstrom et al., 2014) however, our studies suggest that increasing the levels of this hormone acutely can lead to negative affective biases. In humans, higher progesterone levels have been shown to enhance memory for negative emotional images (Ertman et al., 2011) and intrusive recollections of negative emotional events (Ferree et al., 2011).

In the ABT, we observed no effect with testosterone treatment in female rats but a negative bias following acute dosing in males. These findings are opposite to those previously reported for the forced swim test where an antidepressant like effect was observed (Buddenberg et al., 2009). Castrated male rats were also observed to have more depression-like behaviours in this assay suggesting low testosterone levels may be pro-depressant however, there are limitations associated with the forced swim test (McHenry et al., 2014). The antidepressant-like effects of testosterone have been linked to its conversions into oestradiol, as treatment with dihydrotestosterone, a testosterone metabolite that cannot be aromatised to oestradiol, did not induce the same effect (McHenry et al., 2014). However, we previously observed positive biases with oestradiol in the ABT in male rats and therefore, this does not appear to be a mechanism contributing to the effects observed in this assay. We also observed that blocking the effects of testosterone induced a positive bias suggesting these effects were hormone specific. Again, these effects are not generally the same as has previously been reported although aspects of the experimental protocols do differ. For example, neonatal treatment with flutamide has been shown to increase depression and anxiety-like behaviours in rats (Zhang et al., 2010). In contrast, tests evaluating anxiety-like behaviours using an open field test and a modified Vogel’s conflict model, have demonstrated anxiolytic properties following flutamide treatment (Svensson, 2012). In humans, cancer treatments involving androgen blockade have also been linked to adverse effects on cognition and mood (Cherrier et al., 2009). The ability of flutamide treatment to induce positive biases in both sexes may be related to its effects on serum levels of oestradiol (O’Connor et al., 2002). Whilst differences in the assays used or time course of treatment may be relevant, another possible cause of the negative bias in male rats is that treatment may have induced an increase in inter-male aggression which can then cause psychosocial stress. One of the most widely used rodent models of depression is the chronic social defeat model (Rygula et al., 2005) and studies in the affective bias tasks have previously reported negative affective biases in rats exposed to both acute and chronic psychosocial stress (Papciak et al., 2013; Stuart et al., 2013; Hinchcliffe et al., 2017).

We observed a positive affective bias of oxytocin in both male and female rats in the ABT. These data suggest that as well as having effects on social behaviour and in other measures of emotional processing (Neumann and Landgraf, 2012), acute doses of oxytocin can induce positive affective biases. These findings are consistent with other rodent studies where acute oxytocin (Balmus et al., 2018) as well as carbetocin (Broadbear et al., 2014) treatment reduces immobility in the forced swim test in rats. Interestingly, in a judgement bias task in dogs, oxytocin was found to induce a positive affective bias (Kis et al., 2015). Higher levels of oxytocin are a potential biomarker of positive emotion in dogs and humans, and a facilitator of pro-social behaviour in rodents (Kis et al., 2015). A study in humans has also shown that oxytocin can reduce cortisol levels in humans during stress conditions (Cardoso et al., 2013). We also observed positive biases in male but not female rats, when animals were treated with desmopressin. These effects are contrary to previous findings, where anxiogenic effects have been reported (Mak et al., 2012) and treatment with vasopressin antagonists have had antidepressant effects (Iijima et al., 2014). Desmopressin interacts with AVP1 receptors, which would be expected to increase aggressive behaviour and cause psychosocial stress and negative biases. However, we used a very low dose of desmopressin because of its powerful antidiuretic properties. If it could be done safely (e.g. avoiding water retention, low blood sodium and seizures, Verbalis, 1993), use of a higher dose would be desirable to investigate dose dependency of any induced biases. It would also be interesting to test the vasopressin receptor 1 antagonists, which have previously been found to have anxiolytic and antidepressant effects and are selective for the AVP1 receptor thus reducing effects associated with fluid homeostasis.

The ABT is an appetitive task, however, we have previously reported effects for a wide range of compounds which are in a direction that does not consistently relate to changes in motivation or appetite (Stuart et al., 2013, 2015; Hinchcliffe et al., 2017; Stuart et al., 2017). However, previous studies have shown that sex hormone manipulations may affect appetite and food intake (Krishnan et al., 2016). The results from our voluntary consumption test did not find any effects of oestrogen, progesterone or testosterone on appetite when compared to the vehicle group although there was some variation in the data and differences between vehicle and progesterone in absolute consumption per body weight female rats. This finding seems to be the opposite of that observed in previous preclinical studies. Authors have demonstrated that acute progesterone treatment (10 mg/kg) causes an increase in appetite in female mice (Kaur and Kulkarni, 2002). There was also no effect on latencies during the task consistent with a lack of effect on motivation or general locomotor function.

In summary, this study has shown that acute manipulation of gonadal hormone levels using exogenous administration or treatment with antagonists can induce biases in reward learning and memory in the ABT. Both male and female rats were similarly affected by the peptide hormones and female gonadal hormones but with differences in their response to testosterone. There is growing interest in the relationship between affective biases and mood disorders and these data suggest that hormones may also be an important modulator of these biases (Hales et al., 2014; Stuart et al., 2017; Robinson, 2018). Both our studies in rodents and work in humans suggest that there is a direct relationship between the ability of an acute treatment to induce an affective bias and their longer-term impacts on mood (Harmer et al., 2017; Stuart et al., 2017; Robinson, 2018). At this time, we have only looked at the acute effects of these hormones and further studies to investigate chronic treatments and affective biases during fluctuations in hormone levels (e.g. following withdrawal) would also be of interest. The effects of these treatments on the acute and chronic effects of stress (Guo et al., 2018) is also an area of interest and could be further investigated using the ABT and our assay of reward-induced bias, which is sensitive to impairments in a range of putative depression models (Stuart et al., 2017; Robinson, 2018; Stuart et al., 2019).

## Declaration of Competing Interest

The authors have no relevant conflicts of interest to declare. ESJR has received research funding grants from MRC, BBSRC, NC3Rs and Wellcome Trust as well as Boehringer Ingelheim, Eli Lilly, Pfizer and MSD. MM has received research funding as PI from BBSRC, NC3Rs, Royal Society, Defra, UK Home Office, UFAW, RSPCA.
