# Supplementary material for: Investigating hormone-induced changes in affective state using the affective bias test in male and female rats
Source: Psychoneuroendocrinology. 2020 May;115:104647. doi: 10.1016/j.psyneuen.2020.104647 (PMC7193894; doi:10.1016/j.psyneuen.2020.104647)
Supplement: Supplementary file 1 [file mmc1.docx]

**Supplementary materials**

|  | **Substrate ‘A’** | **Substrate ‘B’** | **Substrate ‘Blank’** |
| --- | --- | --- | --- |
| test 1 | felt | shredded dishcloth blue | exfoliating gloves |
| test 2 | absorbent fibre | string | foam shapes |
| test 3 | dusters | tissue paper balls | yellow bath sponge |
| test 4 | black satin | cardboard | rope |
| test 5 | fur | polyester | pompoms |
| test 6 | cellulose sponge | corrugated paper | perlite |
| test 7 | purple ribbon | green raffia ribbon | sparkly pompoms |
| test 8 | brown pet bedding | cork | hessian sack |
| test 9 | cotton wool balls | stringy cloth | hairbands |
| test 10 | organza | silk | shredded paper |
| test 11 | bin liner | plastic scourer | straws |
| test 12 | cotton mix | leather | balloons |
| test 13 | chubby wool | shoe laces | velcro |
| test 14 | brown partition paper | dishcloth squares | polyester lining |
| test 15 | aspen | cypress | coloured matchsticks |
| test 16 | Christmas ribbon | umbrella | tights |
| test 17 | towel | canvas | pipe cleaners |
| test 18 | newspaper | paper pet bedding | confetti |
| test 19 | suede | chenille strands | yellow fleece |
| test 20 | poster squares | polystyrene  polPolystyrene | sequins |
| test 21 | crepe paper squares | scarf yarn | sparkling fibre |
| test 22 | denim | rucksack strap | foam pad |

**Table S1: List of the substrates used in the experiments in both cohorts.**

| **Treatment** | **Dose (mg/kg)** | **Response latency (s)** | | **Trials to criterion** | |
| --- | --- | --- | --- | --- | --- |
|  |  | **Vehicle** | **Drug** | **Vehicle** | **Drug** |
| **Oestradiol** | 0.00 | 1.7±0.1 | 1.6±0.1 | 8.2±0.4 | 8.1±0.4 |
|  | 0.001 | 1.8±0.2 | 1.9±0.2 | 7.9±0.3 | 8.0±0.5 |
|  | 0.01 | 1.6±0.1 | 1.8±0.1 | 8.1±0.2 | 7.8±0.3 |
| **Formestane** | 0.00 | 1.5±0.04 | 1.4±0.04 | 6.5±0.1 | 6.4±0.1 |
|  | 1.00 | 1.5±0.05 | 1.6±0.07 | 6.4±0.1 | 6.4±0.1 |
|  | 10.00 | 1.5±0.05 | 1.4±0.04 | 6.3±0.1 | 6.4±0.1 |
| **Bisphenol A** | 0.00 | 1.7±0.1 | 1.7±0.1 | 6.7±0.2 | 6.7±0.2 |
|  | 0.05 | 1.7±0.1 | 1.7±0.1 | 6.9±0.2 | 6.9±0.3 |
|  | 0.50 | 1.9±0.2 | 1.7±0.1 | 6.9±0.2 | 6.8±0.2 |
| **Progesterone** | 0.00 | 1.6±0.1 | 1.6±0.1 | 6.4±0.1 | 6.7±0.2 |
|  | 1.00 | 1.5±0.0 | 1.6±0.1 | 6.7±0.2 | 6.6±0.2 |
|  | 10.00 | 1.6±0.1 | 1.6±0.1 | 6.6±0.2 | 6.6±0.1 |
| **Testosterone** | 0.00 | 1.9±01 | 1.9±0.1 | 7.2±0.3 | 6.7±0.1 |
|  | 1.00 | 1.8±0.1 | 1.8±0.1 | 6.7±0.1 | 7.0±0.2 |
|  | 10.00 | 1.9±0.1 | 1.8±0.1 | 7.0±0.2 | 6.9±0.2 |
| **Flutamide** | 0.00 | 1.5±0.1 | 1.5±0.1 | 6.4±0.1 | 6.5±0.1 |
|  | 1.00 | 1.6±0.1 | 1.6±0.1 | 6.5±0.1 | 6.5±0.2 |
|  | 10.00 | 1.5±0.1 | 1.5±0.1 | 6.4±0.1 | 6.4±0.1 |
| **Vehicle** | 0.00 | 1.5±0.1 | 1.5±0.1 | 6.4±0.1 | 6.6±0.2 |
| **Carbetocin** | 0.30 | 1.5±0.1 | 1.6±0.1 | 6.5±0.1 | 6.3±0.1 |
| **Desmopressin** | 0.10 | 1.5±0.1 | 1.5±0.1 | 6.5±0.1 | 6.3±0.1 |

**Table S2: Pairing session data following acute hormone manipulations in male rats.** Data shown as mean (n=12 animals/group) ± SEM averaged from the two pairing sessions for each substrate-reward association (control/vehicle or manipulation/drug).

| **Treatment** | **Dose (mg/kg)** | **Response latency (s)** | | **Trials to criterion** | |
| --- | --- | --- | --- | --- | --- |
|  |  | **Vehicle** | **Drug** | **Vehicle** | **Drug** |
| **Oestradiol** | 0.00 | 1.6±0.1 | 1.5±0.1 | 6.5±0.1 | 6.4±0.1 |
|  | 0.001 | 1.5±0.1 | 1.5±0.1 | 6.3±0.1 | 6.2±0.1 |
|  | 0.01 | 1.7±0.1 | 1.6±0.1 | 6.3±0.1 | 6.4±0.1 |
| **Formestane** | 0.00 | 1.4±0.1 | 1.5±0.1 | 6.4±0.1  ± | 6.4±0.1 |
|  | 1.00 | 1.5±0.1 | 1.4±0.0 | 6.5±0.1 | 6.3±0.1 |
|  | 10.0 | 1.4±0.0 | 1.5±0.1 | 6.4±0.1 | 6.4±0.1 |
| **Bisphenol A** | 0.00 | 1.6±0.1  1.0 | 1.7±0.2 | 6.5±0.1 | 6.3±0.1 |
|  | 0.05 | 1.7±0.1 | 1.6±0.1 | 6.2±0.1 | 6.4±0.1 |
|  | 0.50 | 1.6±0.1 | 1.6±0.2 | 6.4±0.1 | 6.3±0.1 |
| **Progesterone** | 0.00 | 1.5±0.0 | 1.5±0.1 | 6.6±0.1 | 6.6±0.1 |
|  | 1.00 | 1.5±0.1 | 1.5±0.1 | 6.6±0.2 | 6.5±0.1 |
|  | 10.00 | 1.5±0.1 | 1.6±0.1 | 6.7±0.1 | 6.4±0.1 |
| **Testosterone** | 0.00 | 1.5±0.1 | 1.5±0.1 | 6.7±0.2 | 6.5±0.2 |
|  | 1.00 | 1.5±0.1 | 1.4±0.1 | 6.6±0.3 | 6.6±0.2 |
|  | 10.00 | 1.4±0.1 | 1.4±0.1 | 6.5±0.2 | 6.8±0.2 |
| **Flutamide** | 0.00 | 1.4±0.0 | 1.5±0.1 | 6.2±0.1 | 6.2±0.1 |
|  | 1.00 | 1.5±0.1 | 1.5±0.1 | 6.3±0.1 | 6.3±0.1 |
|  | 10.00 | 1.5±0.1 | 1.5±0.1 | 6.3±0.1 | 6.2±0.1 |
| **Vehicle** | 0.00 | 1.6±0.1 | 1.7±0.2 | 6.3±0.1 | 6.3±0.1 |
| **Carbetocin** | 0.30 | 1.7±0.1 | 1.7±0.2 | 6.3±0.1 | 6.4±0.1 |
| **Desmopressin** | 0.10 | 1.6±0.1 | 1.6±0.1 | 6.3±0.1 | 6.3±0.1 |

**Table S3: Pairing session data following acute hormone manipulations in female rats.** Data shown as mean (n=12 animals/group) ± SEM averaged from the two pairing sessions for each substrate-reward association (control/vehicle or manipulation/drug).
